# Supplementary material for: Perceptual metacognition of human faces is causally supported by function of the lateral prefrontal cortex
Source: Commun Biol. 2020 Jul 9;3:360. doi: 10.1038/s42003-020-1049-3 (PMC7347936; doi:10.1038/s42003-020-1049-3)
Supplement: Supplementary file 1 — Supplementary Information [file 42003_2020_1049_MOESM1_ESM.pdf]

Supplementary Information

Supplementary Figure 1.

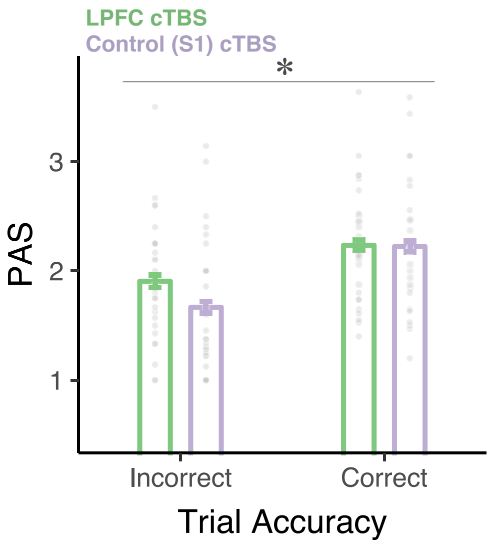

Subjective visibility (PAS) as a function of accuracy and cTBS site following face orientation discrimination obtained at each participant's near-threshold contrast. After cTBS to LPFC, participants reported higher subjective visibility of face stimuli that had been incorrectly identified—thereby reducing estimates of metacognitive awareness. Each dot depicts the data of one participant (N= 28). Error bars represent within-subjects standard errors (1).

Supplementary Figure 2. Stimulus discrimination accuracy (*d'*) and subjective visibility (PAS) are plotted as a function of cTBS for face orientation (**a & b**) and emotion (**c & d**) discrimination tasks at participants' near-threshold contrast. cTBS to LPFC did not reliably change participants' accuracy (**a**) or subjective visibility (**b**) during face orientation discrimination. In the emotion discrimination task, accuracy (**c**) increased slightly following cTBS to LPFC, while subjective visibility (**d**) remained unchanged. Error bars represent within-subjects standard errors (1).

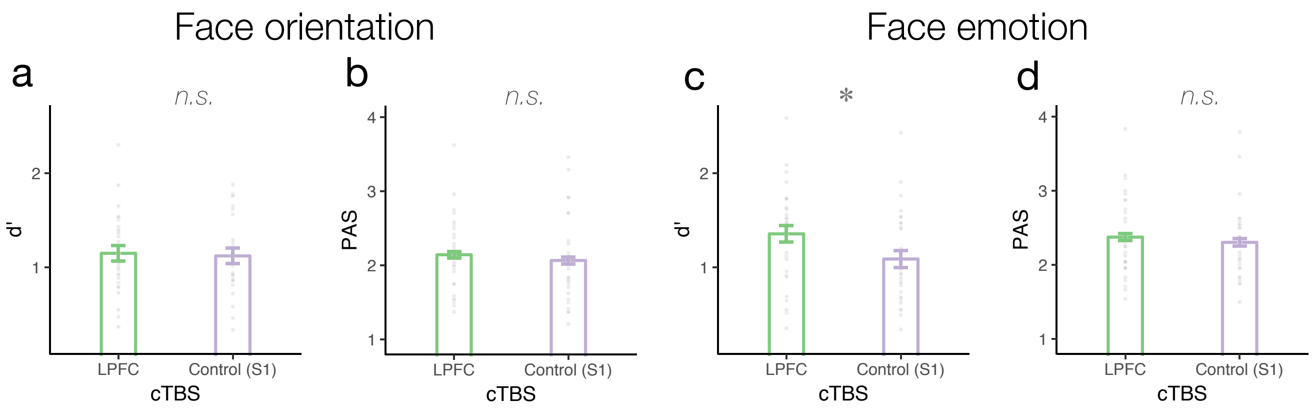

Supplementary Figure 3. Neurosynth derived resting-state functional connectivity maps (Yeo et al. 2011) (N = 1,000 subjects; MNI space, 2mm isotropic) are shown for DLPFC sites targeted in previous TMS studies of metacognitive awareness, thresholded at $r > .2$. While some overlap of functional connectivity fingerprints is observed across studies in parietal cortex, distinct visual-cortical and intra-prefrontal network profiles are noted when comparing Rounis et al. (2010) and the present study vs. Rahnev et al. (2016) and Shekhar & Rahnev (2018).

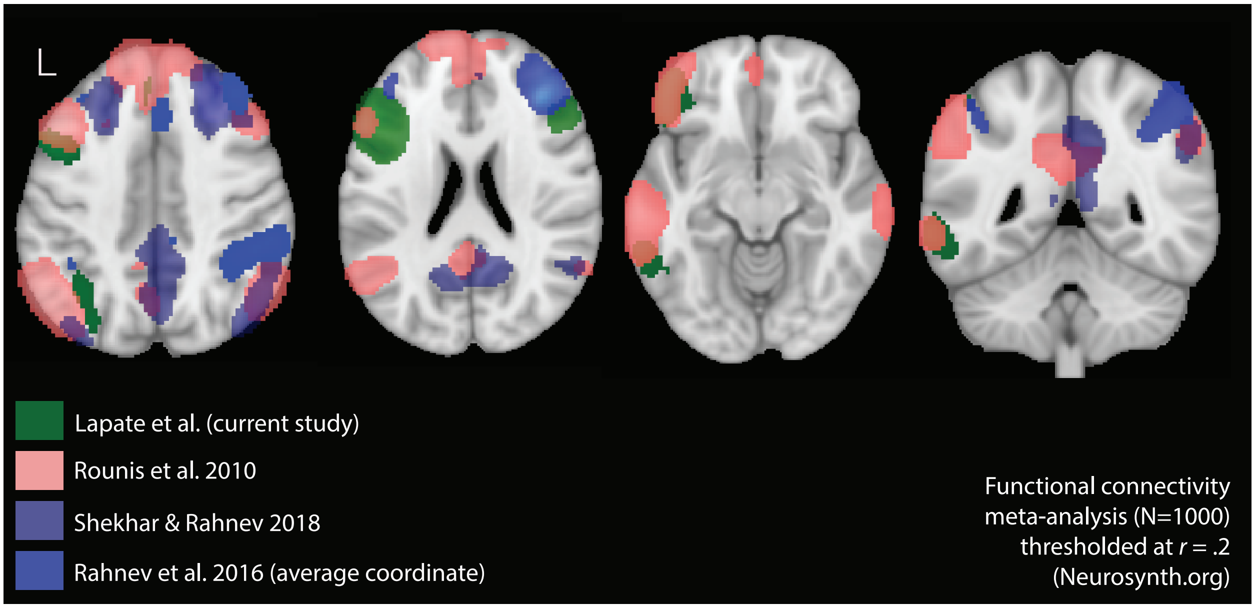

The functional connectivity maps above can be assessed here:
Lapate et al (current study): https://neurosynth.org/locations/-48_24_20_6/
Rounis et al 2010 (left coordinate): https://neurosynth.org/locations/-40_18_52_6/
Shekhar & Rahnev 2018: https://neurosynth.org/locations/28_30_38_6/
Rahnev et al. 2016 (average coordinate): https://neurosynth.org/locations/38_34_28_6/

<u>Supplementary Table 1</u>: *Total number of zero cell counts by cTBS condition (LPFC or S1), stimulus-discrimination accuracy (Correct or Incorrect), and PAS rating (1-4) at participants' near-threshold contrast in the face-orientation task.*

	**Correct Trials**				**Incorrect Trials**			
	1	***2***	***3***	***4***	***1***	***2***	***3***	***4***
**LPFC**	5	1	5	14	9	4	11	22
**S1**	6	0	3	12	5	4	19	25

<u>Supplementary Table 2:</u> *Total number of zero cell counts by cTBS condition (LPFC or S1), stimulus-discrimination accuracy (Correct or Incorrect), and PAS rating (1-4) at participants' near-threshold contrast in the face-emotion task*

	**Correct Trials**				**Incorrect Trials**			
	1	***2***	***3***	***4***	***1***	***2***	***3***	***4***
**LPFC**	16	2	3	16	13	2	17	29
**S1**	11	0	4	13	13	3	17	28

Supplementary Results

The nature of metacognitive impairment following cTBS to LPFC.

We examined the association between subjective visibility ratings and objective stimulus discrimination performance at participants' near-threshold contrast in order to clarify the nature of metacognitive awareness impairment produced by LPFC cTBS. We found that inhibitory cTBS to LPFC increased subjective stimulus visibility after incorrect trials, as indicated by a significant interaction of performance accuracy (correct vs. incorrect) by cTBS site (LPFC vs. Control/S1): W = 7.85, p = 0.009. As shown below in **Supplementary Figure 1**, higher subjective visibility ratings followed incorrect discrimination trials when LPFC was inhibited by cTBS compared to when LPFC was intact.

Analysis of all participants (independently of near-threshold performance).

As detailed in the *Methods*, our analysis included data of participants for whom the method of constant stimuli adequately captured their near-threshold performance (i.e. where the 95% performance CI included 75% for at least one contrast level). For completeness, we also examined whether the results of the impact of LPFC cTBS on face orientation metacognitive awareness held when including all participants in the analysis, regardless of whether their near-threshold performance was captured by the contrasts used in Method of Constant Stimuli. Upon including all participants (including ones whose threshold performance was not captured), we still found that LPFC cTBS attenuated metacognitive efficiency, which is the metacognitive awareness measure that best controls for variation in first-order (stimulus discrimination) task performance, contrast * cTBS interaction meta-*d'* − *d'* W = 18.625, p = .019, η_p^2 = .375]. Effects were not present in other measures of metacognition, Type 2 AUC W = 11.421, p = .117, η_p^2 = .269; meta-d' W = 9.696, p = .172, η_p^2 = .238, further reinforcing the key finding that intact

LPFC function impacts metacognition of complex face stimuli primarily during perceptually ambiguous (i.e. near-threshold) visual experiences.

Interaction of task and cTBS on metacognitive awareness

Although cTBS reduced metacognitive awareness for face orientation and not emotion, the formal test of the difference of the magnitude of the impact of cTBS by task (i.e. cTBS * task interaction) occasionally approached but did not reach two-tailed statistical significance for most metrics, as follows: *At participants' near-threshold contrast*: Type 2 AUC: W = 3.36, p = .077, η_p^2 =.115; meta-*d'*: W = 4.09, p = .053, η_p^2 =.136 ; meta-*d'* − *d'* W = 0.3, p >.59, η_p^2 = .01. *Across contrasts*: Type 2 AUC: W = 3.29, p =.083, η_p^2 = .116; meta-*d'*: W= 3.97, p = .058, η_p^2 = .137; meta-*d'* − *d'* (reported in the main text) W = 5.183, p = .034, η_p^2 = .172.

Reference

1. R. Morey, Confidence intervals from normalized data: A correction to Cousineau (2005). *Tutor. Quant. Methods Psychol.* **4**, 61–64 (2008).
