# Supplementary material for: Perceptual metacognition of human faces is causally supported by function of the lateral prefrontal cortex
Source: Commun Biol. 2020 Jul 9;3:360. doi: 10.1038/s42003-020-1049-3 (PMC7347936; doi:10.1038/s42003-020-1049-3)
Supplement: Supplementary file 2 — Reporting Summary [file 42003_2020_1049_MOESM2_ESM.pdf]

nature research

Corresponding author(s): Regina Carla Lapate

Last updated by author(s): May 28, 2020

Reporting Summary

Nature Research wishes to improve the reproducibility of the work that we publish. This form provides structure for consistency and transparency in reporting. For further information on Nature Research policies, see Authors & Referees and the Editorial Policy Checklist.

Statistics

For all statistical analyses, confirm that the following items are present in the figure legend, table legend, main text, or Methods section.

n/a	Confirmed	
☐	☒	The exact sample size (*n*) for each experimental group/condition, given as a discrete number and unit of measurement
☐	☒	A statement on whether measurements were taken from distinct samples or whether the same sample was measured repeatedly
☐	☒	The statistical test(s) used AND whether they are one- or two-sided<br>*Only common tests should be described solely by name; describe more complex techniques in the Methods section.*
☒	☐	A description of all covariates tested
☐	☒	A description of any assumptions or corrections, such as tests of normality and adjustment for multiple comparisons
☐	☒	A full description of the statistical parameters including central tendency (e.g. means) or other basic estimates (e.g. regression coefficient) AND variation (e.g. standard deviation) or associated estimates of uncertainty (e.g. confidence intervals)
☐	☒	For null hypothesis testing, the test statistic (e.g. *F*, *t*, *r*) with confidence intervals, effect sizes, degrees of freedom and *P* value noted<br>*Give P values as exact values whenever suitable.*
☒	☐	For Bayesian analysis, information on the choice of priors and Markov chain Monte Carlo settings
☒	☐	For hierarchical and complex designs, identification of the appropriate level for tests and full reporting of outcomes
☐	☒	Estimates of effect sizes (e.g. Cohen's *d*, Pearson's *r*), indicating how they were calculated

Our web collection on statistics for biologists contains articles on many of the points above.

Software and code

Policy information about availability of computer code

Data collection	PsychoPy 2 (v. 1.79.01) run in Python.
Data analysis	For statistical analysis: R Studio Version 1.0.153. R version 3.4.1. Packages: tidyr_0.7.2; dplyr_0.7.4; reshape2_1.4.2; ggplot2_2.2.1; MANOVA.RM_0.3.2; binom_1.1-1. For computation of metacognitive awareness metrics: https://github.com/metacoglab/HMeta-d/blob/master/Matlab/fit_meta_d_mcmc.m for d', meta-d' & meta-d'-d'; https://github.com/metacoglab/meta_dots/blob/master/type2roc.m for Type 2 ROC/AUC.

For manuscripts utilizing custom algorithms or software that are central to the research but not yet described in published literature, software must be made available to editors/reviewers. We strongly encourage code deposition in a community repository (e.g. GitHub). See the Nature Research guidelines for submitting code & software for further information.

Data

Policy information about availability of data

All manuscripts must include a data availability statement. This statement should provide the following information, where applicable:

- Accession codes, unique identifiers, or web links for publicly available datasets
- A list of figures that have associated raw data
- A description of any restrictions on data availability

Experimental materials (code written in PsychoPy) have been made publicly available via Open Science Framework and can be accessed at https://osf.io/t8m4j/. Institutional Review Board constraints at the University of Wisconsin-Madison (Health Sciences) precluded the authors from publicly sharing the raw data. The raw data are being stored at a secure server at the University of Wisconsin-Madison. All source data underlying the figures (Fig. 2a-f, Fig. 3a-d, Fig. 4a-f, Sup Fig. 1 and Sup Fig. 2a-d) are available via the Open Science Framework and can be accessed at: https://osf.io/dgmz9/.

Field-specific reporting

Please select the one below that is the best fit for your research. If you are not sure, read the appropriate sections before making your selection.

☐ Life sciences ☒ Behavioural & social sciences ☐ Ecological, evolutionary & environmental sciences

For a reference copy of the document with all sections, see nature.com/documents/nr-reporting-summary-flat.pdf

Behavioural & social sciences study design

All studies must disclose on these points even when the disclosure is negative.

Study description

The data are experimental (within-subjects design) and quantitative.

Research sample

We recruited thirty-four right-handed individuals from the University of Wisconsin–Madison between the ages of 18-35 y who had been previously screened in a clinical interview for neurological and psychiatric conditions, as well as for TMS and MRI safety criteria. One participant did not tolerate TMS delivered to the LPFC target, therefore rendering the maximum sample size N = 33 (19 males; age 18-32; M = 23.79, SD = 4.428). The sample is representative of the required healthy sample for TMS+MRI studies.

Sampling strategy

Sampling was random. The sample size for the present study was determined based on a power analysis performed on data from the most pertinent published experiment probing the causal role of LPFC function on visual metacognition via cTBS that was available at the time of participant recruitment (Rounis et al. 2010). In their study, statistical power obtained for the paired-mean difference of metacognitive awareness sensitivity following cTBS to LPFC vs. sham was d = 0.693, which required a sample size of n = 19 to detect a statistically significant effect at alpha two-tailed p < .05 and power = 80%.Therefore, with the goal of retaining a minimum of n = 19 participants with usable data across the multiple (n = 2) TMS sessions and (n = 4) psychophysical assessments, we recruited thirty-four right-handed individuals from the University of Wisconsin–Madison.

Data collection

Prior to behavioral data collection, a high resolution MRI was collected from the participants to allow for accurate neuronavigation.

During behavioral data collection, participants sat at with their eyes positioned 80cm away from the computer monitor (ASUS HDMI set to 60Hz refresh rate; 53cm screen width; 1920 x 1080 pixels resolution). At least n=2 researchers were present during TMS administration and data collection (RCL and one or more undergraduate research assistants). The experiment was single blind (RCL was not blind to the experimental hypothesis; undergraduate research assistants were).

TMS was delivered to the left LPFC and to medial S1 with a Magstim Super Rapid magnetic stimulator (Magstim, Whitland, UK) equipped with a figure-8 stimulating coil. Precise TMS targeting on a subject-by-subject basis was achieved via a Navigated Brain Stimulation (NBS) system (Nextstim, Helsinki, Finland), which uses infrared-based frameless stereotaxy to map the position of the coil and the subject's head in relation to the space of the individual's high-resolution MRI. In order to temporarily interfere with function of LPFC and Control/S1 sites, we used a continuous TMS protocol—cTBS—consisting of 50Hz trains of 3 TMS pulses repeated every 200 ms continuously over a period of 20 seconds (300 pulses total). As is typical with this TMS protocol, we delivered cTBS at 80% of active motor threshold.

Timing

Start date: December 2014. End date: May 2015.

Data exclusions

We used the method of constant stimuli to determine psychophysical performance and estimate metacognition for near-threshold stimuli (as per prior work and a primary goal of the present manuscript), as well as to examine the potential role of LPFC in promoting metacognition outside of the near-threshold range. We report in the main manuscript analysis of participants whose data allowed for the appropriate estimation of their stimulus detection threshold for each metacognition task, as detailed next. For the face orientation task, n=28 (out of 33) participants' stimulus-detection threshold fell within the contrast range spanned by the stimuli used in the method of constant stimuli; those participants were therefore included in the analysis (n=5 participants were at ceiling; final sample: 16 males; age 18-32; M = 23.5, SD = 4.718). In the emotional-expression discrimination task, n=32 (out of 33) met this criterion (n=1 participant was at floor; final sample: 19 males; age 18-32; M = 23.81, SD = 4.497)—those participant groups therefore comprise the final sample used for data analysis in the main manuscript. Results with all participants included (regardless of whether their near-threshold performance was captured) are reported as a Supplementary Result.

Non-participation

n=1 participant did not tolerate TMS administration to their LPFC target.

Randomization

The design is within-subjects--all subjects underwent all experimental conditions. Assignment to "LPFC site first" vs. "S1 site first" was based on subject number (odd or even). Assignment of whether awareness task of face orientation vs. face emotion was tested first was based on session number in an orthogonal manner to the TMS site order assignment (where awareness task order alternated every n=4 sessions).

Reporting for specific materials, systems and methods

We require information from authors about some types of materials, experimental systems and methods used in many studies. Here, indicate whether each material, system or method listed is relevant to your study. If you are not sure if a list item applies to your research, read the appropriate section before selecting a response.

Materials & experimental systems

n/a	Involved in the study
☒	☐ Antibodies
☒	☐ Eukaryotic cell lines
☒	☐ Palaeontology
☒	☐ Animals and other organisms
☐	☒ Human research participants
☒	☐ Clinical data

Methods

n/a	Involved in the study
☒	☐ ChIP-seq
☒	☐ Flow cytometry
☐	☒ MRI-based neuroimaging

Human research participants

Policy information about studies involving human research participants

Population characteristics	See above.
Recruitment	Participants (18-35 y old) were recruited using a mass e-mail to students at UW Madison. Thus, the population sampled may be considered above-average on years of education relative to the general population. It is unlikely but unknown whether educational achievement may impact estimates of metacognitive awareness or susceptibility to TMS procedures.
Ethics oversight	University of Wisconsin-Madison

Note that full information on the approval of the study protocol must also be provided in the manuscript.

Magnetic resonance imaging

Experimental design

Design type	N/A (only structural)
Design specifications	N/A (only structural)
Behavioral performance measures	N/A (only structural)

Acquisition

Imaging type(s)	structural (T1-weighted)
Field strength	3T
Sequence & imaging parameters	MRI data were acquired with a 3.0 T GE scanner (GE Healthcare, Waukesha, WI) using an 8-channel coil. High-resolution 3D T1-weighted inversion recovery fast gradient echo (Mugler, 1990) anatomical images were collected in 160 contiguous 1.25 x 1.25 x 1.25-mm sagittal slices (TE = 2.3 ms; TR = 5.6 ms; flip angle = 12°; FOV = 240 x 240 mm; 192 x 192 x 160 data acquisition matrix, inversion time TI = 450 ms).
Area of acquisition	Whole brain.

Diffusion MRI ☐ Used ☒ Not used

Preprocessing

Preprocessing software	We used FSL version 5.0.7. FSL's Bet was used for skull stripping. Structural data were reoriented to RPI using Afni's 3dresample.
Normalization	The structural (T1-weighted) data were registered to the MNI152 template to allow for the back-projection of the MNI coordinate for the LPFC site into each subject's native space; FSL's FLIRT was used for that purpose: https://fsl.fmrib.ox.ac.uk/fsl/fslwiki/FLIRT
Normalization template	MNI152 template (used for back projection of an MNI group coordinate (Lapate et al. 2016) into each subject's native space).
Noise and artifact removal	N/A (only structural data)
Volume censoring	N/A (only structural data)

Statistical modeling & inference

Model type and settings	N/A (only structural data)

Effect(s) tested	N/A (only structural data)

Specify type of analysis: ☐ Whole brain ☐ ROI-based ☐ Both

Statistic type for inference (See Eklund et al. 2016)	N/A (only structural data)
Correction	N/A (only structural data)

Models & analysis

n/a	Involved in the study
☒	☐ Functional and/or effective connectivity
☒	☐ Graph analysis
☒	☐ Multivariate modeling or predictive analysis
